# Effects of social comparison on variety-seeking behavior: the roles of lack of perceived control and self-reflection

**DOI:** 10.3389/fpsyg.2025.1534275

**Published:** 2025-03-31

**Authors:** Cheng Che, Yuxin He, Miaomiao Zhou

**Affiliations:** School of Economics and Management, China University of Petroleum (East China), Qingdao, China

**Keywords:** social comparison, lack of perceived control, variety-seeking, self-reflection, compensatory consumption

## Abstract

**Background:**

Social comparison, especially upward social comparison, has always been a common concern and experience in people’s lives. Although social comparison and its consequences have been extensively studied in previous literature, few scholars have paid attention to its influencing mechanism and boundary conditions on the compensatory consumption of variety-seeking.

**Objective:**

This study used experimental methods to investigate whether social comparison is related to variety-seeking behavior, and to explore the mediating role of lack of perceived control and the moderating role of self-reflection.

**Methods:**

In total, 414 participants were recruited for 3 experiments. They were divided into groups to complete their respective experimental tasks, including social comparison manipulation, variety-seeking manipulation, self-reflection manipulation, and lack of perceived control scale.

**Results:**

The results of the three experimental studies showed that compared with downward-comparison individuals, upward ones presented higher variety-seeking tendencies and the lack of perceived control mediated between the two, and that was moderated by self-reflection. Specifically, in the absence of self-reflection, upward comparisons will result in a higher propensity for variety-seeking behavior compared to downward ones. However, after engaging in self-reflection, the effect was no longer significant, while the mediating effect no longer held.

**Conclusion:**

The research highlights the relationship between social comparison and variety-seeking behavior, reveals the mediating mechanism between them, and deepens the understanding of how upward social comparison increases variety-seeking behavior. The above research results have positive significance for promoting the theory of social comparison, lack of perceived control and variety-seeking behavior, guiding the marketing practice of enterprises, and helping individuals reduce the negative impact of upward comparison.

## 1 Introduction

Social comparison is a common phenomenon in daily life, encompassing areas such as academic rankings, occupational income and prestige, clothing brands and prices, and physical attractiveness. People strive to build a more objective self-perception framework, using it as a basis for self-orientation and future planning. Due to differences in reference points, social comparisons often lead to varying degrees of psychological pressure and emotional fluctuations. In general, individuals have an upward drive to “expect themselves to be better than others” and therefore tend to compare themselves with those who are perceived as better (Buunk and Gibbons, 2007). However, this tendency can also lead to a range of negative consequences. As a result, finding ways to mitigate these negative effects has become a key area of academic focus.

In this context, consumption behavior is seen as having a potential role in psychological adjustment and self-expression. Among the various forms of consumption, compensatory consumption has garnered significant attention. One such form, variety-seeking behavior, which is characterized by the search for new consumption options or a range of products and services, plays a crucial role in both personal and business development. Specifically, for many consumers, variety-seeking is not only essential for personal growth and adapting to change (Foxall, 1993), but also adds a “flavor” to life, contributing to personal uniqueness and fulfilling emotional needs. For businesses, this behavior helps cultivate a distinctive brand image and drives demand. It also promotes balanced sales across various product categories and encourages consumers to try new or unfamiliar products (Zhang, 2022). Therefore, understanding the factors that influence variety-seeking behavior is of significant value.

Previous research on variety-seeking behavior has primarily focused on external environmental factors (Yang et al., 2024; Lu et al., 2024; Sun et al., 2023). In recent years, however, attention has gradually shifted toward individual-level factors, such as exploring the role of perceived power (Wang and Jin, 2022). Despite this shift, there is still a lack of research on the influence of external group references and self-evaluation in the context of variety-seeking behavior. Specifically, when individuals experience psychological pressure from social comparison, can variety-seeking behavior provide psychological comfort and compensation? What is the internal mechanism underlying this process? These are the central questions of this study.

Self-reflection is an major research object in the field of management psychology. According to the social cognitive theory, self-reflection plays an important role in individual thinking dispersion and experience accumulation (Hao et al., 2016). However, the current research on this variable in academia is mainly focused on the sociological and psychological level (Xu et al., 2022; Pena and Losada, 2017), with little in-depth analysis of its utility in the consumer domain.

Based on the above considerations, this paper primarily approaches the issue from the perspective of compensatory consumption, with a focus on analyzing the underlying mechanisms and boundary conditions of social comparison and variety-seeking behaviors. The goal is to enrich the theoretical frameworks of social comparison theory, social cognitive theory, and other related, while offering new insights into the psychological mechanisms of compensatory consumption. Additionally, it aims to assist businesses in gaining a deeper understanding of consumers’ internal needs, thereby enabling more targeted and effective marketing strategies.

## 2 Literature reviews and hypothesis development

### 2.1 Social comparison

Social comparison refers to the social cognitive process in which individuals form self-assessment by comparing themselves with others in terms of wealth level, academic achievement, social status and other dimensions. Based on the difference in the direction of comparison, it can be divided into three categories: upward comparison, parallel comparison and downward comparison (Festinger, 1954). Among them, upward comparisons are relatively more common in social situations, which refers to comparing with individuals whose situation is better than one’s own.

Social comparison is likely to cause changes in an individual’s psychological state, which in turn affects subsequent consumption behavior. Specifically, upward social comparison generates two types of effects: assimilation and contrast effects (Mussweiler, 2001). The assimilation effect is manifested as individuals’ firm belief that they can achieve the same status as the reference objects. Under such effect, individuals will take the reference objects as their goal for progress and make continuous efforts, with the expectation of achieving self-improvement. The contrast effect, on the other hand, shows that individuals will question their own ability and value, lower their self-assessment (Tesser et al., 1988), and even trigger anxiety, depression (McCarthy and Morina, 2020; Li, 2019) or other negative emotions.

In light of the differences in individual traits and the diversity of stage-specific needs, individuals in this situation tend to choose different consumption patterns. For those with a strong desire for self-expression or a longing for group belonging, they generally reshape their social identities through uniqueness seeking (Gong and Zhang, 2020), status consumption (Liang et al., 2024) and conspicuous consumption (Xu et al., 2023). When they are in urgent need of emotional catharsis, they may be inclined to release through impulsive and compulsive consumption (Liu et al., 2019). In addition, they may also show a relatively high preference for products emphasizing ability (Zheng et al., 2022).

Downward social comparison, on the other hand, usually stimulates positive emotions and leads to an increase in their self-esteem levels (Morse and Gergen, 1970). Generally speaking, individuals in this context may have a greater need for warm objects (Zheng et al., 2022) and prefer minimalist consumption (Chen et al., 2024).

In addition, both upward and downward comparison individuals have the intention to buy green products, which is mediated by self-threat and pride, respectively (Wang et al., 2025).

### 2.2 Variety-seeking behavior

Variety-seeking behavior is a manifestation of consumer behavior in which consumers become dissatisfied with the status quo based on individual characteristics, social pressures, product boredom, etc., and shift their consumption choices or seek out new consumption options to achieve stimulus-seeking and bring them new satisfactions (Caracciolo et al., 2022). This behavior is specifically shown in actions like switching between products, categories and brands, or the number of different products or services selected from a fixed choice set in a given choice scenario (Kahn et al., 1986). Therefore, the measurement of this behavior is usually based on the number of different product or service choices, and the number of switches. The greater the number of choices or transitions between different items, the stronger the variety-seeking tendency of the consumer.

In response to the behavior generating motives, Mcalister and Pessemier (1982) broadly categorized them into two types of motives: derived and direct motives. Existing studies have explored all of these motivations to some extent, mainly centred around environmental factors [e.g., time change (Kelley et al., 2019), environmental monotony (Jin, 2024)], decision factors [e.g., choice timing (Simonson, 1990)], product factors [e.g., size of choice set (Liu et al., 2015)], subjective and objective product types (Baltas et al., 2017) and other perspectives developed. In terms of personal values, relevant studies have mainly dealt with political ideology (Lu et al., 2024), goal pursuit (Rafieian et al., 2024) etc. There is a relative lack of relevant research on the level of self-worth perception, and no clear conclusion has been drawn. Therefore, this study takes it as an entry point to explore, hoping to provide new perspectives and insights for this research field.

### 2.3 Social comparison and variety-seeking behavior

The social comparison process affects individuals’ evaluation of their own traits and abilities (Pillai and Nair, 2021). Specifically, individuals who experience upward comparisons will perceive a gap between themselves and their referents, that is they are inferior to others, which can lead to self-threat (Zheng et al., 2022). When an individual encounters a threat to his or her self-concept, competence, or value, he or she will have a strong desire to escape from the negative situation (Sheldon and Kasser, 2008). In general, improving one’s competence in the relevant area is seen as the most direct and fundamental way to solve the problem, but it takes a long time and is difficult to implement, so individuals may turn to consumption behaviors to instantly satisfy such needs or to divert their attention, such as seeking compensatory consumption (Gronmo, 1988). It’s worth noting that variety-seeking is a typical type of such consumption (Zhang, 2022).

In terms of variety-seeking itself, there is an hedonic value inside (Baumgartner and Steenkamp, 1996). In social comparison contexts, threatened individuals experience new stimuli by changing their choices and consumption habits, actively exploring and experimenting with new or diverse products or services, and focusing more attention on the new stimuli (Sood and Drèze, 2006), thus reduce their concern for the threat (Gronmo, 1988). Meanwhile, the experiential values such as satisfaction and pleasure felt from experiencing and acquiring something new and varied (Sood and Drèze, 2006) also help individuals to withdraw from the disillusionment that comes from comparison with others.

In the general situation of comparisons and pressures, variety-seeking such as updating clothes and accessories, tasting multi-flavoured food, and experiencing new lifestyles have become effective ways to divert attention, reduce threats and pressures, and bring new pleasurable feelings to individuals (Zhang, 2022) Therefore, individuals engaged in upward social comparison are more inclined to enjoy such positive experiences; that is, they tend to seek diverse choices. On the contrary, downward comparative individuals are less likely to feel threatened and hardly question themselves, so they hardly show a significant preference for variety-seeking behavior.

Based on this, the following hypotheses are proposed in this paper:

*H1:* Individuals encountering upward comparison have a higher propensity to engage in variety-seeking behavior compared to downward ones.

### 2.4 The mediating role of lack of perceived control

Sense of control refers to an individual’s perception of his or her ability to significantly influence events in the environment (Burger, 1989). It has become one of the most important factors influencing people’s behavioral decisions. Scholar such as Adler point out that human have an innate desire for controlling over their environment, thus acquiring and maintaining a high sense of control is a basic need (Averill, 1973). People’s preference for control stems primarily from the opportunity it provides to test their abilities and the increased likelihood that desired outcomes will be realized.

However, events that lead to a reduced sense of control occur in daily life, such as natural disasters, social factors, and psychological factors. Among them, it has been pointed out that, there is a consistency between lack of control and perceived self-discrepancy (Cutright and Samper, 2014). Individuals facing self-threats will feel uncertain about themselves and a sense of uncontrollability towards the surrounding environment (Campbell et al., 2003). As a result, the individual’s sense of self-control is diminished in self-threatening situations.

According to the control compensation theory, when control is threatened, there is a strong desire to restore control. Recovering methods always fall into two main categories: firstly, through one’s own efforts to improve competence and strengthen personal beliefs, which is a challenging process; and secondly, indirectly seeking external support such as compensatory consumption (Landau et al., 2015). Variety-seeking behavior is seen as a symbol of free choice and autonomy, through which individuals exert control and mastery over their environment, thereby enhancing their sense of personal control (Wang et al., 2022; Stephens et al., 2007).

Based on this, the following hypotheses are proposed in this paper:

*H2:* Lack of perceived control mediates between social comparison and variety-seeking behavior.

### 2.5 The moderating role of self-reflection

Based on the social cognitive theory perspective, individual’s self-modelling is not solely influenced by the external environment, but can be actively realized through a series of cognitive mechanisms. Specifically, individuals can actively participate in the formation and deepening of self-knowledge through intentional thinking, self-regulation and self-reflection (Locke, 1987), and adjust and optimize their own cognitive structure and behavioral patterns.

Among them, self-reflection is an in-depth process of self-examination and cognition, which is manifested in the continuous, positive, repeated and careful examination, evaluation and understanding of one’s own way of thinking mode, behaviors, emotional expressions, and problems encountered by the individual (Grant et al., 2002). Scholars have proposed that self-reflection is conducive to individual psychological adjustment and has a positive impact on the suppression of negative cognition and emotions (Takano and Tanno, 2009), and individuals who are good at self-reflection usually have a more optimistic attitude towards their own failure (Jones et al., 2009).

Therefore, in the context of upward comparisons, self-reflective individuals are able to consider the gap between them and their referents from a more objective, positive and optimistic perspective, and reflect on the unfavourable comparative situation due to their own subjective reasons, such as the inappropriate setting of the comparison object, self-imposed limitations and weak self-drive. In this way, they will counteract the negative cognitive impacts that may be generated by the behavior of pure social comparisons. Consequently, the motivation to seek emotional catharsis for stress, dissatisfaction, etc. tends to diminish, which in turn may obviate the need to seek compensation through variety-seeking as a consumption way. In contrast, individuals in downward comparison situations generally do not develop threat perceptions, and thus self-reflection still has no impact on their consumption tendencies.

Based on this, the following hypotheses are proposed in this paper:

*H3a:* Self-reflection moderates the effect of social comparison on variety-seeking behavior. Specifically, without self-reflection, upward comparisons led to a higher propensity for variety-seeking behavior; after self-reflection, there was no significant difference in the effect of social comparisons on variety-seeking behavior.

*H3b:* Self-reflection moderates the mediating role of lack of perceived control between social comparison and variety-seeking behavior. Specifically, the mediating role of lack of perceived control is present when self-reflection is not engaged in, and it is absent when self-reflection is engaged in.

**Figure 1:**
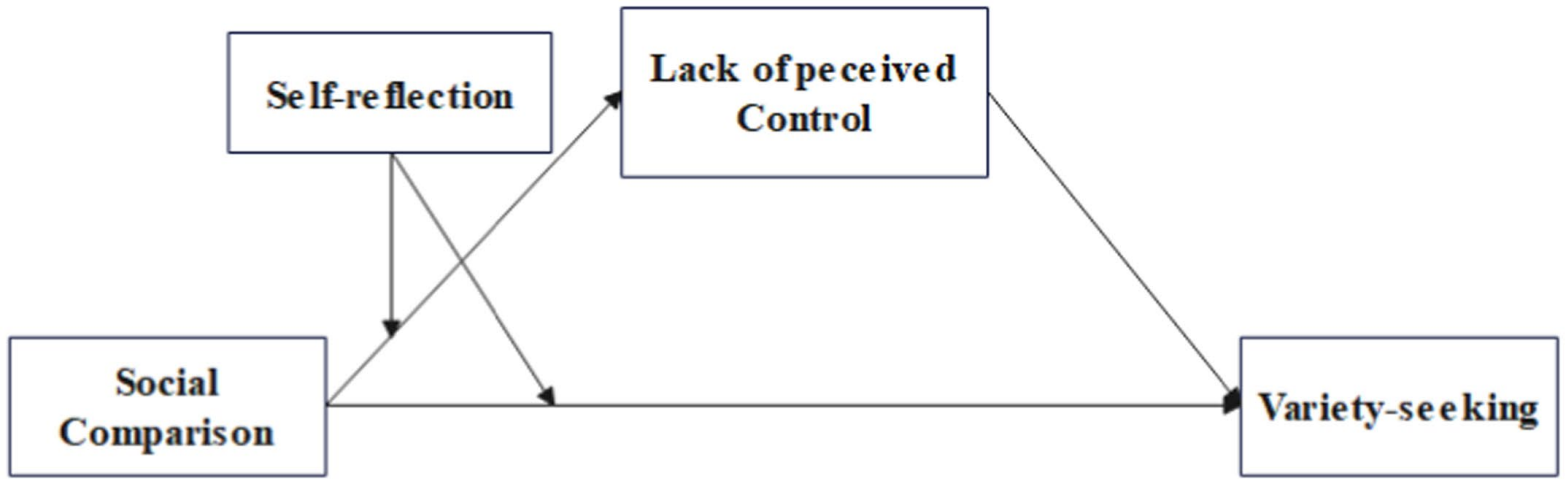
Conceptual model.

The theoretical framework of this study is shown in Figure 1.

## 3 Materials and method

### 3.1 Experiment 1

#### 3.1.1 Subjects and preparation

Experiment 1 was used to test the effect of social comparison on variety-seeking behavior, i.e., to validate H1. 101 experimental participants were from a university in Shandong province, of which 52 were males and 49 were females, with an average age of 22.88 years. All participants were randomly and evenly assigned into (social comparison: upward vs. downward) groups, of which 51 were in the upward comparison group and 50 were in the downward comparison group.

#### 3.1.2 Design

The experiment was conducted under the name of “Life Decision-making Survey.” The manipulation of social comparison draws on Schlosser and Levy's (2016) writing task. After the recall and writing tasks were completed, subjects’ responses to the question regarding “To what extent do you consider your current situation to be superior to that of the person you are describing.” are measured on a 7-point Likert scale (1 = very little, 7 = very much).

Then, they moved on to another investigation allegedly unrelated to the aforementioned task. Participants were asked to imagine buying candies at a convenience store, and pictures of the six flavors were displayed on a large screen in front of the classroom. The flavors are blueberry, cola, chocolate, banana, strawberry and orange respectively, and there was no difference except for color. Later, they were told that they needed to buy a total of 5 candies, during which they could mix and match any flavors, and wrote the number of candies they wanted to buy in the questionnaire. After that, they submitted the questionnaire to the field organizer. The number of different candy flavors they chose will be used as a basis for measuring variety-seeking behavior. The more candy flavors they chose, the higher their tendency towards variety-seeking. Finally, the participants filled in their personal information and were paid accordingly.

#### 3.1.3 Analysis and results

Data analyses began with a dummy variable for the independent variable social comparison, i.e., the upward comparison group was coded as 1, and the downward comparison group was coded as 0. Afterwards, a social comparison manipulation test was conducted using an independent sample *t*-sample. The result showed that the upward comparison group scored significantly lower than the downward comparison group, i.e., the upward comparison group had a more unfavourable perception of their own status quo (M_up_ = 3.86, SD_up_ = 1.114, M_down_ = 4.84, SD_down_ = 1.017, t(99) = −4.601, *p* < 0.01), revealing the manipulation of social comparison was successful. Next, main effects analyses showed that compared to the participants in the downward comparison group, the participants in the upward comparison group significantly presented a higher variety-seeking tendency (M_up_ = 4.04, SD_up_ = 1.166, M_down_ = 3.28, SD_down_ = 1.325, t(99) = 3.059, *p* < 0.01). The results of the independent samples *t*-test are shown in Table 1. Hypothesis 1 is verified.

**Table 1:** Results of independent samples *t*-test for Experiment 1.

Variable		*F*	*t*	Sig.(two-tailed)
Perceived better	Assumed equal variance	1.792	−4.601	0.000
Status quo	Not assumed		−4.605	0.000
Variety-seeking	Assumed equal variance	2.178	3.059	0.003
	Not assumed		3.055	0.003

### 3.2 Experiment 2

#### 3.2.1 Subjects and preparation

Experiment 2 tested the mediating role of the lack of perceived control by changing the variety-seeking experimental stimuli, i.e., testing H2. 129 participants were recruited to participate in this experiment through the Credamo platform, including 65 males and 64 females, with an average age of 33.08 years. Upon entering the laboratory, all participants were randomly and equally assigned to groups, including 64 in the upward comparison group and 65 in the downward comparison group.

#### 3.2.2 Design

The experiment was a one-way (social comparison: upward vs. downward) between-groups design. The social comparison was manipulated in the same way as in Experiment 1, followed by the question “To what extent do you think that the person you are describing is better than you are” (1 = very little, 7 = very much). Upon completion, participants were asked to complete the lack of perceived control scale (Michinov, 2005), which consisted of 12 items such as “I cannot do just about anything I really set my mind to” and was scored on a 7-point Likert scale (*α* = 0.803).

This was followed by the variety-seeking behavior test. The situational prompt was: “Coincidentally, the store you pass by is having a promotion. There are 5 flavors of ice cream available in the freezer for you to choose from, namely vanilla, raspberry, grape, matcha, and sea salt. The prices are the same and the supply is sufficient. You can choose 4 ice creams with the same or different flavors, just as you like.” The number of flavors served as the basis for the behavior of seeking variety. Finally, the participants answered demographic questions and received the rewards.

#### 3.2.3 Analysis and results

Manipulation tests were first conducted by independent samples *t*-tests. Compared with the downward comparison group, the upward comparison group significantly felt that the reference object was superior to them (M_up_ = 4.73, SD_up_ = 1.300, M_down_ = 3.95, SD_down_ = 1.556, t(127) = 3.090, *p* < 0.01), which indicates that the social comparison manipulation is valid. The perceived level of control loss was later measured in both groups. The results showed that participants in the upward comparison group also perceived a stronger sense of control loss compared to ones in the downward comparison group (M_up_ = 4.55, SD_up_ = 0.693, M_down_ = 4.14, SD_down_ = 0.627, t(127) = 3.532, *p* < 0.01).

Secondly, after coding for variety-seeking, a main effects analysis was conducted. The data indicated that participants in the upward comparison group presented a higher variety-seeking tendency than those in the downward comparison group (M_up_ = 3.27, SD_up_ = 1.011, M_down_ = 2.58, SD_down_ = 1.059, t(127) = 3.733, *p* < 0.01). The results are shown in Table 2.

**Table 2:** Results of independent samples *t*-test for Experiment 2.

Variable		*F*	*t*	Sig. (two-tailed)
Perceived better	Assumed equal variance	1.244	3.090	0.002
Status quo	Not assumed		3.094	0.002
Lack of perceived control	Assumed equal variance	2.883	3.532	0.001
	Not assumed		3.529	0.001
Variety-seeking	Assumed equal variance	0.387	3.733	0.000
	Not assumed		3.735	0.000

Then, mediated effect analysis was carried out with social comparison direction as the independent variable, lack of perceived control as the mediating variable and variety-seeking as the dependent variable. The PROCESS programme Model 4 was chosen and the results are displayed in Table 3. Results show that the mediating effect result of the lack of perceived control does not contain 0 (LLCI = 0.0107, ULCI = 0.2871), and the effect value is 0.1137. It indicates that social comparison will affect individual variety-seeking behavior through the lack of perceived control, and thus H2 is verified.

**Table 3:** Results of the mediation effect test for the lack of perceived control in Experiment 2.

	Effect	SE	LLCI	ULCI
Total effect	0.6810	0.1824	0.3201	1.0420
Direct effect	0.5673	0.1889	0.1935	0.9412
Indirect effect	0.1137	0.0690	0.0107	0.2871

### 3.3 Experiment 3

#### 3.3.1 Subjects and preparation

Experiment 3 tested the moderating effect of self-reflection based on changing the social comparison manipulation, i.e., testing H3a and H3b. 184 participants were recruited to participate in this experiment, including 90 males and 94 females, with a mean age of 29.41 years. This experiment adopted a 2 (social comparison: upward vs. downward) × 2 (self-reflection: yes vs. no) between-groups design. After the participants entered the laboratory, they were randomly assigned, with 46 people in each group.

#### 3.3.2 Design

The manipulation of social comparison in this experiment drew on Gong and Zhang (2020), with the question “To what extent do you feel your current situation is worse than the person you describe?” (1=very little,7=very much). Upon completion of the above experiment, the manipulation experiment of self-reflection was entered, which was adapted from Steinrücke et al. (2023), the recall method used. Subsequently, the participants completed the lack of perceived control scale (*α* = 0.887) and the variety-seeking behavior manipulation. Above method and content were almost the same as in Experiment 2, with the exception of the selection number: Experiment 3 required participants to select 5 ice creams from 5 flavors. Finally, participants completed basic personal information and were paid for their participation.

#### 3.3.3 Analysis and results

Tests of the validity of the manipulation were first carried out. The results of the independent samples *t*-test showed that the upward comparison group scored higher than the downward comparison group, i.e., the comparison objects were perceived to be significantly better off in terms of economic level and material conditions than participants were (M_up_ = 5.33, SD_up_ = 1.563, M_down_ = 4.32, SD_down_ = 1.617, t(182) = 4.312, *p* < 0.01), indicating that the social comparison manipulation was successful. In addition, lack of perceived control perceived by participants in the upward comparison group was also more intense compared to ones in the downward comparison group, as reflected in the measurements in both groups (M_up_ = 4.35, SD_up_ = 0.918, M_down_ = 3.62, SD_down_ = 1.046, t(182) = 5.039, *p* < 0.01). Secondly, their variety-seeking behavior was analyzed. The results showed that the upward comparison group scored significantly higher in variety-seeking tendency and presented higher variety-seeking behaviors compared to the downward comparison subjects (M_up_ = 3.84, SD_up_ = 1.082, M_down_ = 3.15, SD_down_ = 1.099, t(182) = 4.259, *p* < 0.01). Relevant results are shown in Table 4.

**Table 4:** Results of independent samples *t*-test for Experiment 3.

Variable		*F*	*t*	Sig. (two-tailed)
Perceived better	Assumed equal variance	0.965	4.312	0.000
Status quo	Not assumed		4.312	0.000
Lack of perceived control	Assumed equal variance	0.193	5.039	0.000
	Not assumed		5.039	0.000
Variety-seeking	Assumed equal variance	0.023	4.259	0.000
	Not assumed		4.259	0.000

**Figure 2:**
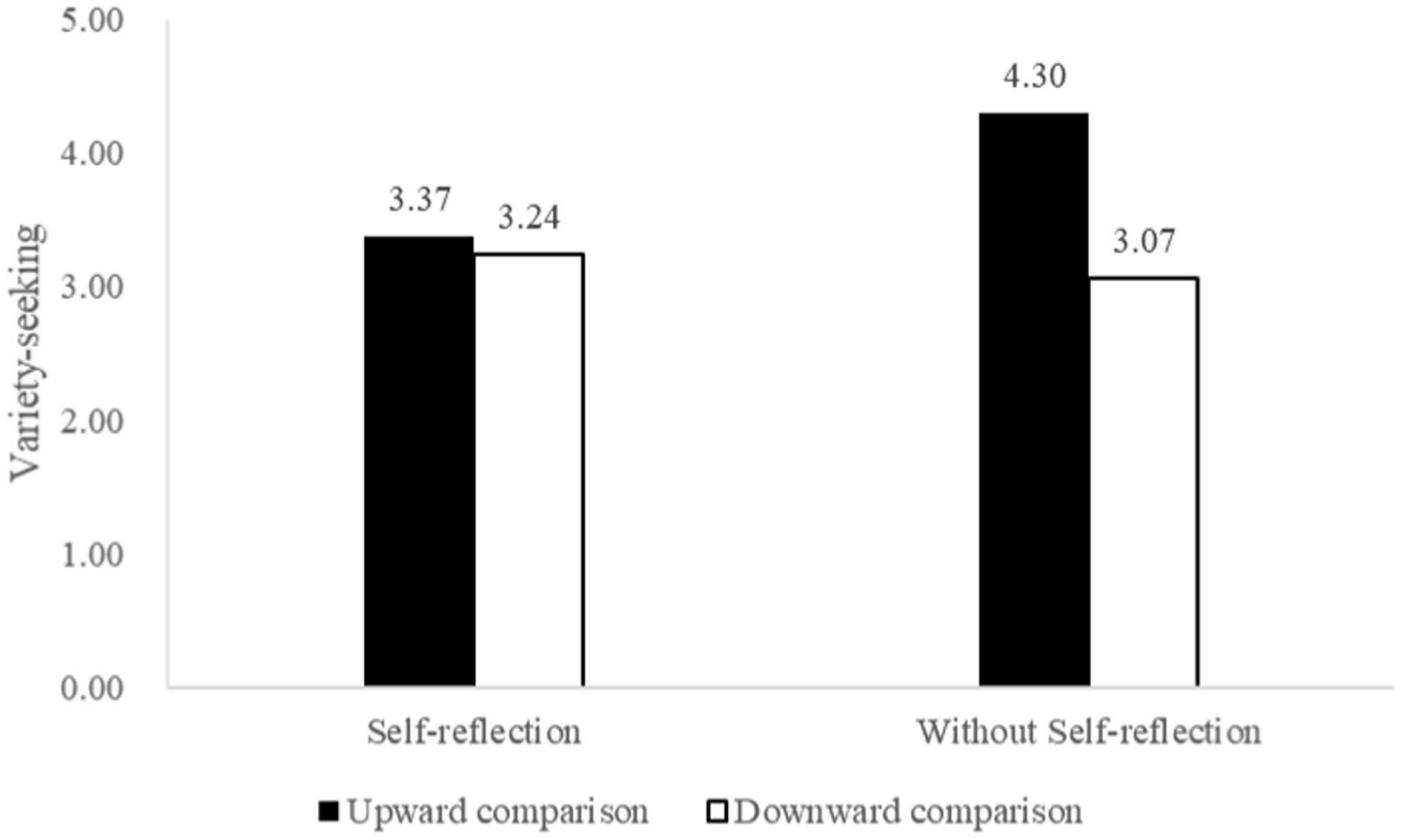
Interactive effects of social comparison and self-reflection on variety-seeking behavior.

Next, a multifactor ANOVA was conducted with social comparison (upward vs. downward), self-reflection (yes vs. no) and their interaction terms as the independent variables and variety-seeking as the dependent variable. Results of the test are presented in Table 5. The data showed a significant main effect of social comparison (*F*(1,180) = 19.847, *p* < 0.001), self-reflection (*F*(1, 180) = 6.126, *p* < 0.05), and an interaction between the two (*F*(1, 180) = 13.006, *p* < 0.001). Subsequently, simple effects analyses were conducted. The results indicated that without self-reflection, the upward comparison group had a significantly higher variety-seeking tendency than the downward comparison group (M_up_ = 4.30, M_down_ = 3.07, *F*(1,180) = 32.494, *p* < 0.01). After conducting self-reflection, two groups did not show a significant difference in variety-seeking tendencies (M_up_ = 3.37, M_down_ = 3.24, *F*(1,180) = 0.360, *p* = 0.549), as shown in Figure 2.

**Table 5:** Tests for between-subjects effects.

Variable	Variance	Degrees of freedom	Mean square	*F*	Sig.	Biased Eta square
Social comparison	21.571	1	21.571	19.847	0.000	0.099
Self-reflection	6.658	1	6.658	6.126	0.014	0.033
Social comparison × Self-reflection	14.136	1	14.136	13.006	0.000	0.067
Error	195.630	180	1.087			
Total	2486.000	184				

Model 8 in the PROCESS was then selected to carry out the test of the moderated mediation effect. The data showed a significant interaction between social comparison and self-reflection on lack of perceived control (*β* = −0.6069, SE = 0.2841, *t* = −2.1362, *p* < 0.05, LLCI = −1.1675, ULCI = −0.0463), as well as between social comparison and self-reflection on variety-seeking (β = −1.0026 SE = 0.3081, *t* = −3.2546, *p* < 0.01, LLCI = −1.6105, ULCI = −0.3947). Upon further analysis, it was found that the mediating effect of the lack of perceived control in the pathway of the influence of social comparison on variety-seeking was significant in the context of no self-reflection (LLCI = 0.0045, ULCI = 0.4231), with an effect value of 0.1808, and that, after self-reflection, the results of the test for the mediating effect of the lack of perceived control included 0 (LLCI = −0.0021, ULCI = 0.1734), and the mediating effect was not significant. Combining the results of these analyses, H3a and H3b were validated.

Experiment 3 verified the moderating effects of self-reflection between social comparison and variety-seeking as well as the moderating effect on the mediating effect of lack of perceived control. Specifically, in the no self-reflection context, upward comparisons had a significant positive effect on variety-seeking and the mediating effect of the lack of perceived control was present. However, when in a self-reflective situation, these effects were no longer significant.

## 4 Results and discussion

### 4.1 Results

Our research systematically explored the influence mechanism and boundary conditions between social comparison and variety-seeking based on social comparison theory and social cognitive theory. Through three formal experiments, we tested four hypotheses proposed. Experiment 1 indicated that individuals in upward (vs. downward) comparison group had a higher tendency to engage in variety-seeking behavior no longer significant. Main effect was again verified in different types of comparison contexts and variety-seeking stimuli. Experiment 2 investigated the mediating role of lack of perceived control, demonstrating that people who underwent upward comparison would sense a loss of control and consequently seek more varieties. In Experiment 3, the moderating effect of self-reflection was confirmed. Specifically, upward comparisons led to a higher propensity for variety-seeking behavior when self-reflection was not involved, yet once it was conducted, the effect became insignificant. In addition, self-reflection also moderated the mediating impact of lack of perceived control. In the absence of self-reflection, this mediating effect is observable. However, following self-reflection, it was no longer significant.

### 4.2 Theoretical contributions

This research mainly has the following three theoretical contributions. To begin with, different from the existing social comparison studies, which mostly focus on symbolic and emotional cathartic consumption (Xu et al., 2023; Liu et al., 2019), this study pays attention to the quantity of type selection, namely variety-seeking. Both direct and indirect effects are confirmed, indicating that a relatively new outcome variable of social comparison and internal mechanisms are revealed, and the conclusion that variety-seeking can be a compensatory consumption mode is confirmed once again.

In addition, our study takes social comparison as the key antecedent variable affecting variety-seeking, expanding the application scope of variety-seeking behavior in the external group reference and self-evaluation dimension, and promoting the academic circle’s deeper understanding of this behavior. Previous studies have shown that variety-seeking behavior is mainly affected by decision-making environment, product information and etc. (Wang et al., 2023; Bandyopadhyay, 2024), but less attention is paid to personal traits and interpersonal factors. In this research, such factors are included to be explored, which helps scholars understand variety-seeking behavior more deeply and comprehensively, and provide new ideas and directions for subsequent related research.

Moreover, this research innovatively incorporates self-reflection as a boundary condition into the theoretical framework of social comparison and variety-seeking, and extends it from the field of traditional psychological emotion regulation to the marketing decision-making context of compensatory consumption, contributing to the in-depth analysis of consumers’ complex psychology and consumption behavior.

### 4.3 Practical contributions

Based on the above findings, enterprises can integrate a certain degree of social comparison elements into their marketing activities to stimulate consumers’ pursuit of diversified choices. For example, typical social comparison scenarios such as comparison of academic performance and wealth levels can be presented in the promotional slogans in the sales area. By emphasizing the values of “Diverse choices, enriching colourful life” and “Myriad styles, a brand-new chapter,” and placing a variety of products in conspicuous sales areas to promote sales, consumers can be influenced. Meanwhile, marketers can also help consumers make transactions as quickly as possible by limiting the number of promotions, setting a time limit for activities, and providing a quick and easy purchase process, which consciously reduces the time for individual self-reflection and promotes the sale of a variety of products.

In addition, marketers can also identify group characteristics and carry out diversified product marketing for those showing a higher tendency to make upward comparisons or are vulnerable to psychological disadvantages. For example, big data technology can be used to capture and analyze the instant behavior and psychological dynamics of social platform users. When the data show that users frequently make upward comparisons during a specific period, marketers can recommend diversified products to them in order to meet consumers’ desire to seek psychological balance and change the status quo; for specific groups such as people with lower socioeconomic status or those in the financial and business circles, marketers should also emphasize the psychological value of diversified products and carry out the publicity and promotion of related products.

### 4.4 Limitations

Although this paper has provided some insights into the relevant variables and their interrelationships, there are still some shortcomings and areas that deserve further exploration. First, the paper concludes that upward social comparison leads to a lack of perceived control, which in turn triggers variety-seeking behavior. This leads to the question of whether a lack of perceived control may also have a similar effect on other consumption behaviors, especially compensatory consumption; second, the model may also be affected by other boundary conditions, such as the type of comparison object, the presence of others, and personality traits such as self-construal. Future research could conduct a more in-depth research based on these perspectives.

## Data Availability

The original contributions presented in the study are included in the article/Supplementary material, further inquiries can be directed to the corresponding author.
